# MED26/421: The Images Component of a Web-based Orthodontic Patient Records System

**DOI:** 10.2196/jmir.1.suppl1.e66

**Published:** 1999-09-19

**Authors:** D Xenakis, A Vergari, V Vold, N Morlandsto

**Affiliations:** ^1^University of BergenNorway

**Keywords:** Medical Records, Diagnostic Imaging, Computers, Databases

## Abstract

**Introduction:**

This presentation will focus on the images component of a web-based database system-OrthoLINE-that is developed within the EU-Socrates project "OrthODL", through a collaboration of the orthodontic departments of the universities of Bergen, Gothenburg, Munich and Thessaloniki.

**Methods:**

High costs and various problems associated with the use of traditional images on film have lead several orthodontic departments to consider a shift to digital imaging even though this may entail considerable investments in equipment and training. The work processes that range from the acquisition and archiving to the retrieval of digital images for self-learning, teaching and research include several distinct steps. General principles apply, but different protocols are followed according to the source of images (usually scanner or digital camera), their purpose/future use, and the current digital image archiving and communication specifications and resources. Electronic storage space requirements typically have a major bearing on many of the choices related to the way of handling digital images.

Results;

OrthODL project has sought to strike a balance between cost and benefit of producing, storing and communicating quality images for teaching and learning. OrthODL has also developed guidelines, applicable to several aspects of handling images, that currently are being used and evaluated in Bergen. For instance, guidelines for code-naming image files have been elaborated. Apart from taking care of the storage of images, OrthoLINE system makes possible searches for images in a way that appears to cover adequately the need of instructors to update existing educational material or compose new.

**Discussion:**

Special attention will be given in this presentation to the first experiences with a professional digital camera at the orthodontic department of Bergen, highlighting the perceived advantages and disadvantages of digital- as compared to traditional photography. Speed of data retrieval over the Internet, as well as security issues that arise once image files become part of the medical communication will be further discussed.

